# MiR-543 promotes tumorigenesis and angiogenesis in non-small cell lung cancer via modulating metastasis associated protein 1

**DOI:** 10.1186/s10020-020-00175-1

**Published:** 2020-05-14

**Authors:** Dawei Wang, Li Cai, Xudong Tian, Wenjun Li

**Affiliations:** 1 Department of Thoracic Surgery, Yantaishan Hospital, Yantai, 264000 Shandong China; 2 Department of Pathology, The Affiliated Yantai Yuhuangding Hospital of Qingdao University, Yantai, 264000 Shandong China; 3 Department of Thoracic Surgery, Liaocheng People’s Hospital and Liaocheng Clinical School, No. 67 Dongchang West Road, Liaocheng, 252000 Shandong China; 4 Department of Thoracic Surgery, The Affiliated Yantai Yuhuangding Hospital of Qingdao University, No. 20 Yuhungding East Road, Zhifu District, Yantai, 264000 Shandong China

**Keywords:** miR-543, Non-small cell lung cancer, MTA1, Tumorigenesis, Angiogenesis

## Abstract

**Objective:**

This study is aimed to explore the role of miR-543 in non-small cell lung cancer (NSCLC), and verify whether miR-543 targets metastasis associated protein 1 (MTA1) to affect tumorigenesis and angiogenesis in NSCLC.

**Methods:**

Firstly, miR-543 mimic and inhibitor were transfected into A549 cells and H1299 cells. The cells proliferation was tested by MTT and clone formation. The cells apoptosis was analyzed by cytometry. Tube formation assay was used to measure the vascularization of cells. qRT-PCR and Western Blot were used to measure the MTA1 expression. Dual-luciferase assay was used to analyze whether miR-543 targets MTA1. Secondly, MTA1 mimic and inhibitor were transfected into cells to analyze the effect of MTA1 on proliferation and angiogenesis in NSCLC cells. Lastly, the nude mice were used to verify the effect of miR-543 on tumorigenesis and angiogeneisis in NSCLC via modulating MATA1.

**Results:**

miR-543 overexpression could apparently promote cells proliferation and angiogeneisis in NSCLC cells. Meanwhile, the MTA1 expression was increased after transfecting miR-543 mimic. Dual luciferase reporter assay revealed MTA1 was a downstream target of miR-543. Further studies showed that inhibition of MTA1 weakened the role of miR-543 overexpression in NSCLC cells. Vivo experiments revealed that miR-543 promoted cells proliferation and angiogenesis in tumor tissues via modulating MTA1.

**Conclusion:**

miR-543 could target MTA1 to promote tumorigenesis and angiogenesis in NSCLC via targeting MTA1.

## Background

As the leading cause of cancer deaths, the incidence rate and mortality rate of lung cancer are increasing, which poses a great threat to people’s health and life (Siegel et al. 2017). Clinically, about 80–85% of lung cancers are non-small-cell lung cancers (NSCLCs) (Zhang et al. 2015). However, the estimated overall survival is just 16–17% with distant metastasis in NSCLCs. Therefore, finding effective and accurate molecular targets for distant metastasis of NSCLC are the key to improve the prognosis.

MicroRNAs (miRNAs) are a class of non-coding RNA molecules encoded by endogenous genes with a length of about 22 nucleotides (Guiot et al. 2019). miRNAs have been revealed to play a crucial role in the tumorigenesis of lung cancer. Research on miR-543 has recently become an interested item. It has been found to be abnormally expressed in a number of tumors, such as gastric cancer, oral squamous cell carcinoma, etc. (Li et al. 2016a; Zhao et al. 2018). Overexpression or knockdown miRNAs would cause the corresponding changes in the phenotype of cancer cells. But the effect of miR-543 in NSCLC has not been accurately verified.

It has been confirmed that metastasis associated protein 1 (MTA1) is a downstream target of miR-543 in colorectal cancer (Fan et al. 2016). As a transcriptional coregulator, MTA1 achieves goals by altering the acetylation state of the target protein and its interaction with other molecules (Ma et al. 2017). In recent years, MTA1 has been revealed to promote tumor progression and distant metastasis (Kumar and Wang 2016). In NSCLC, the overexpression of MTA1 could lead to tumor growth and microvessel density (MVD) increase by inducing other relevant oncogenes expression (Ma et al. 2017). However, the interacted effect of miR-543 on MTA1 in NSCLC has not been accurately reported.

In this study, the function of miR-543 on proliferation and vascularization in NSCLC cells was analyzed. Furthermore, the exact relationship between miR-543 and MTA1 was explored to elaborate the mechanism of miR-543 on progression of NSCLC.

## Materials and methods

### Cell culture

Human non-small cell lung cancer cells H1299 (BNCC100859) and A549 (BNCC337696), (ATCCCRL-9609), HUVEC (BNCC337616) were cultured in RPMI-1640 (GIBCO, Invitrogen, USA) added with 10% fetal bovine serum and 1% penicillin (100 U/ml)/streptomycin (100 μg/ml). All of them were incubated in incubators (Thermo, USA) under 37 °C and 5% CO_2_. Logarithmic phase was optimal for further study.

### Cell transfected and classification

In order to study the role of miR-543 on NSCLC cells, five groups were divided, including control group (without any treatment), miR-543 mimic group (transfected miR-543 mimic vector, miR-543 mimic), mimic-NC group (transfected mimic- negative control vector, mimic-NC), miR-543 siRNA group (transfected miR-543 siRNA vector, miR-543 siRNA), siRNA-NC group (transfected siRNA-negative control vector, siRNA-NC).

To further verify the interaction between miR-543 and MTA1 on proliferation and vascularization in NSCLC cells, another six groups were divided: control group, miR-543 mimic group, MTA1 mimic group (transfected MTA1 mimic vector, MTA1 mimic), MTA1 mimic NC group (transfected MTA1 mimic negative control vector, Mm-NC), miR-543 mimic+ MTA1 siRNA group (transfected miR-543 mimic and MTA1 siRNA vector, miR-543 + Ms), miR-543 mimic+ MTA1 siRNA-NC group (transfected miR-543 mimic and MTA1 siRNA negative control vector, miR-543 + Ms-NC).

The vectors were transfected to A549 and H1299 cells by LipofectamineTM2000 (Invitrogen, Carlsbad, CA, USA). After 72 h, qRT-PCR was applied to evaluate transfected efficiency. MiR-543 mimic, miR-543 siRNA, MTA1 mimic, MTA1 siRNA, mimic-NC and siRNA-NC were got from Shanghai GenePharm Pharmaceutical Technology Co., Ltd. (Shanghai, China).

### Quantitative real time polymerase chain reaction (qRT-PCR)

TRIzol (Takara, Dalian, China) was applied to extract RNA. Reverse transcription kit (Applied Biosystems, Waltham, MA, USA) was applied to transcript RNA into cDNA. Mastercycler® nexus X2 (Eppendorf, Hamburg, Germany) was applied to conduct qRT-PCR. The procedure is 95 °C 15 s, 95 °C 15 s, 60 °C 60s (35 cycles). The results were processed by 2^-ΔΔCt^ method. U6 and GAPDH are internal references of miR-543 and MTA1, respectively. The sequences of primers were showed in Table 1.

**Table 1:** The sequence of primers in Table 1

Name	Sequences
MiR-543	F:5′-CCAGCTACACTGGGCAGCAGCAATTCATGTTT-3′ R:5′-CTCAACTGGTGTCGTGGA-3′
U6	F:5′-CTCGCTTCGGCAGCACA-3′ R:5′-AACGCTTCACGAATTTGCGT-3’
MTA1	F: 5’-GACCAGGCAGGCTTTCTATC-3′ R: 5′-CTGTTGATGGGCAGGTAGG-3’
GAPDH	F:5’-AGGTCCACCACTGACACGTT-3′ R: 5′-GCCTCAAGATCAGCAAT-3’

### MTT assay

The cells were collected and adjusted to 1 × 10^4^/mL. The cells were seeded in 96-well plates and put into incubator under 5% CO_2_, 37 °C. After 24 h, 48 h and 72 h, one of the groups was added with 20 μL 5 mg/mL MTT solution. Then it was put into an incubator under 37 °C. After 4 h, the liquid was carefully removed. Then 200 μL DMSO was added. After blending fully, cells of each well were measured optical density value (OD) on the enzyme linked immunoassay (Bio-Rad Laboratories) at 490 nm.

### Plate clone assay

The cells in each group were digested to single-cell suspension. After adjusting concentration, 2 mL suspension in each group was seeded in 6-well plates with 500 cells/well. The cells were dispersed by shaking the culture plates and cultured in an incubator under 37 °C, 5% CO_2_ and saturated humidity. When visible clones appeared in the plates, stopped culture and discarded culture medium. The cells were washed carefully twice by PBS and fixed for 15 min by methyl alcohol. After that, the cells were stained by Giemsa for 10–30 min, washed slowly by water and dried at 37 °C. The number of clones was counted directly by eyes.

### Flow Cytometry

The cells were digested and centrifuged (× 800 g, 5 min), then washed with pre-cold PBS twice. The cells (1 × 10^6^/mL) were suspended by 250 μL 1× binding buffer, then added 5 μL Annexin-V marked FITC for 3 min. 10 μL propidium iodide (PI, 20 μg/mL) was added and mixed for 10 min without light. Before detecting, 400 μL 1× binding buffer was added and mixed. Flow cytometer (Gallios; Beckman Coulter, Inc., Brea, California, USA) was used to detect and Cell Quest software (BD Biosicences, San Diego, CA, USA) was used to analyze.

### Tube formation assay

Matrigel (50 μL, BD Bioscience, USA) was horizontally seeded into 96-well plates and solidified at 37 °C for 0.5 h. HUVECs and tumor cells were collected at 1000 rpm for 5 min. The supernate from each group was used to re-suspend cells. Cells (4 × 10^4^ cells/well) were seeded in matrigel and cultured at 37 °C, 5% CO_2_ for 6 h. Olympus inverted microscope (CKX40, Olympus, NewYork, NY) was used to observe the entire tubes.

### Western blot

The expression of MTA1 protein in each group was detected by western-blot. The primary antibodies were Anti-MTA1 Antibody (1:2000, ab71153, Abcam, UK) and beta-actin polyclonal Antibody (1:1000, ab8227, Abcam, UK). The second antibody was goat anti-rabbit Ig G (1:2000, ab6721, Abcam, UK) which needed to incubate for 1 h. After washing, protein bands were made color by ECL for 3-5 min. Image J (NIH) software was used for gray scale scanning and quantification.

### Dual luciferase reporter assay

The wild type and mutant 3 ‘UTRs of MTA1 were amplified in the pGL3/ luciferase vector (Promega, Madison, WI, USA) and cloned into the downstream of luciferase gene. According to the instructions, luciferase activity of the cells was detected by dual luciferase reporter system (Promega) at 48 h post transfection.

### Xenografts in nude mice

The animal experiment scheme is approved by the animal protection and use committee of Yantaishan Hospital, which follows NIH guidelines (NIH Pub. No. 85–23, revised 1996). Thirty Balb/c nude mice (20 ± 2 g) (Charles river, Beijing, China, SCXK (Jing)20,160,006) were randomly divided into 5 groups (*n* = 6): Model group, miR-543 mimic group, miR-543 siRNA group, MTA1 mimic group and miR-543 mimic+MTA1 siRNA (miR-543 mimic + Ms) group. The transfected A549 and H1299 (3 × 10^6^ cell/mL) from each group were subcutaneously implanted in the left axils of nude mice. Untreated cells were used as control (Model). After 7 days, the tumor size was measured by Vernier calipers every week. And the changes of tumor volume within 1 month were observed. Average tumor volume (mm^3^) = (length × width^2^) /2. After 20 days, 3 nude mice were selected randomly from each group and sacrificed by cervical dislocation after anesthesia with 0.3% pentobarbital sodium (45 mg/kg). Then the tumors were weighed.

### Terminal deoxynucleotidyl transferase-mediated dUTP-biotin nick end labeling (TUNEL)

Paraffin sections (5 μm) were dewaxed with xylene, and hydrated with ethanol. After washing with PBS, In Situ Cell Death Detection Kit (Roche, Shanghai, China) was used for TUNEL staining. Briefly, the reaction mixtures were prepared and added to the samples at 37 °C for 1 h without light before washing with PBS for 3 times. DAB (Maixin Biotech, China) was used for coloring and Beyotime Biotechnology (China) was used for re-dyeing. The sections were dehydrated by ethanol, cleared by xylene and sealed by neutral gum. The apoptotic cells were observed by optical microscope and photographed.

### Immunohistochemistry

Tumor tissues were fixed with 4% paraformaldehyde (Beijing solarbio biotechnology co., LTD.). The sections (4 μm) were dewaxed as usual. After inactivating endogenous peroxidase, the sections were heated to repair antigen. The primary MTA1 antibody (1:200, ab71153, Abcam) and Ki-67 antibody (1:200, ab15580, Abcam) were cultured at 4 °C for overnight. After washing with PBS, biotin-labeled MTA1 secondary antibody (1:800, 85–9043, HRP, Broad Spectrum) was added for 1 h. DAB was used for coloring and Beyotime Biotechnology was used for re-dyeing. Results were observed with an optical microscope.

### Microvascular density (MVD)

CD31 immunohistochemistry in tumor vessels was used to calculate MVD. High-vascular density area (hot-spot) was selected under 100 magnification and counted the MVD values under 400 magnification. The MVD values were analyzed by Weidner counting method (Foote et al. 2005).

### Statistics

SPSS 19.0 was used for data processing. The results were expressed as mean ± standard deviation (^−^X ± SD). ANOVA was used for data analysis among multiple groups following Turkey test. *P* < 0.05 was recognized as statistically significant.

## Results

### miR-543 overexpression promoted cells proliferation of A549 and H1299

**Fig. 1:**
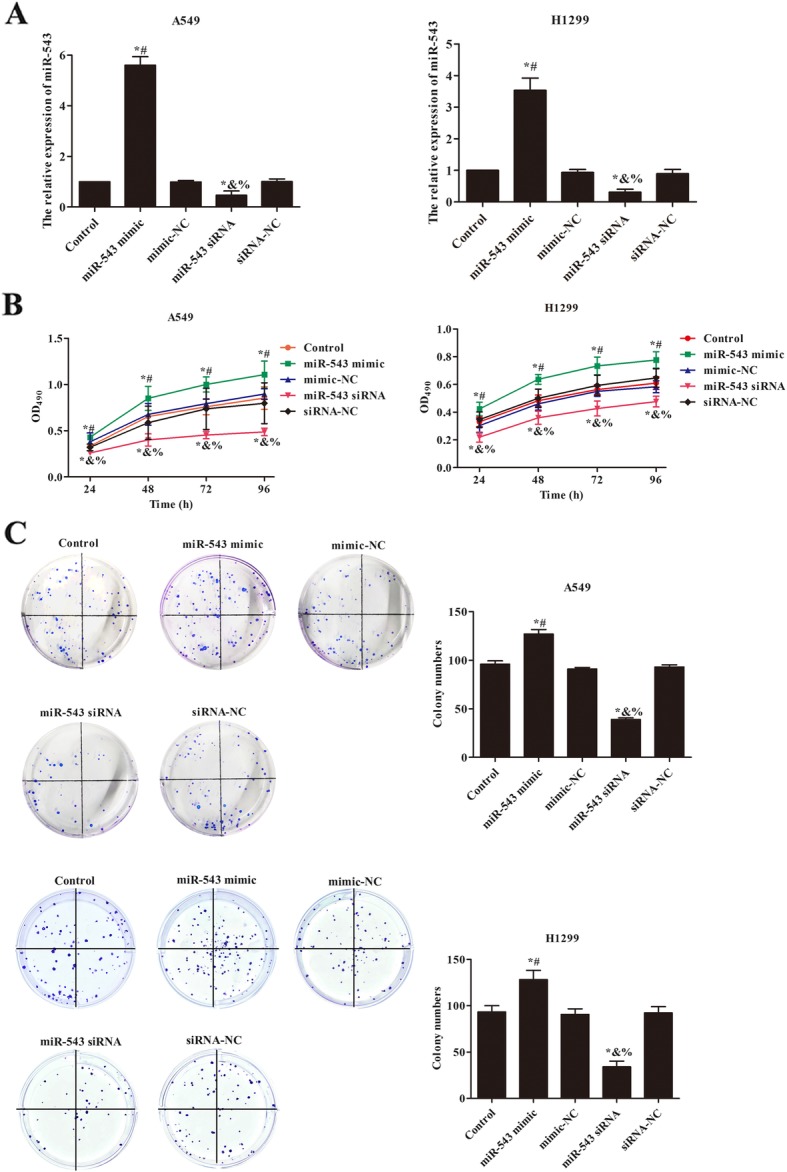
miR-543 overexpression increased the proliferation of A549 and H1299 cells. **a** The relative expression of miR-543 in A549 and H1299 was measured by qRT-PCR. **b** MTT was used to detect the effect of miR-543 on the proliferation of A549 and H1299 cells. **c** Cloning formation assay verified the effect of miR-543 on the proliferation of A549 and H1299 cells. Compared with control group, ^*^p < 0.05; Compared with mimic-NC, ^#^*p* < 0.05; Compared with siRNA-NC, ^&^*p* < 0.05; Compared with miR-543 mimic, ^%^*p* < 0.05

The expression of miR-543 in each group was showed in Fig. 1a. After transfection of miR-543 mimic or miR-543 siRNA, the miR-543 expression in A549 and H1299 cells was statistically up-regulated or down-regulated when compared with control group (*p* < 0.05). After transfection of mimic-NC or siRNA-NC, there was no significant difference compared with control group (*p* > 0.05). Further to observe the effect of miR-543 on cells proliferation in A549 and H1299 cells, the results of MTT were showed in Fig. 1b. Compared with control group, the cells activities of A549 were obviously raised in miR-543 mimic group (*p* < 0.05), while the cell activities of A549 were markedly reduced in miR-543 siRNA group (*p* < 0.05). Similarly results were also showed in H1299 cells. For the results of clone assay (Fig. 1c), the colony numbers significantly increased in miR-543 mimic group contrast to control group (*p* < 0.05), but the colony numbers notably decreased in miR-543 siRNA group (*p* < 0.05). These data suggested that miR-543 overexpression increased the proliferation of NSCLC cells.

### miR-543 overexpression promoted the angiogenesis of A549 and H1299

**Fig. 2:**
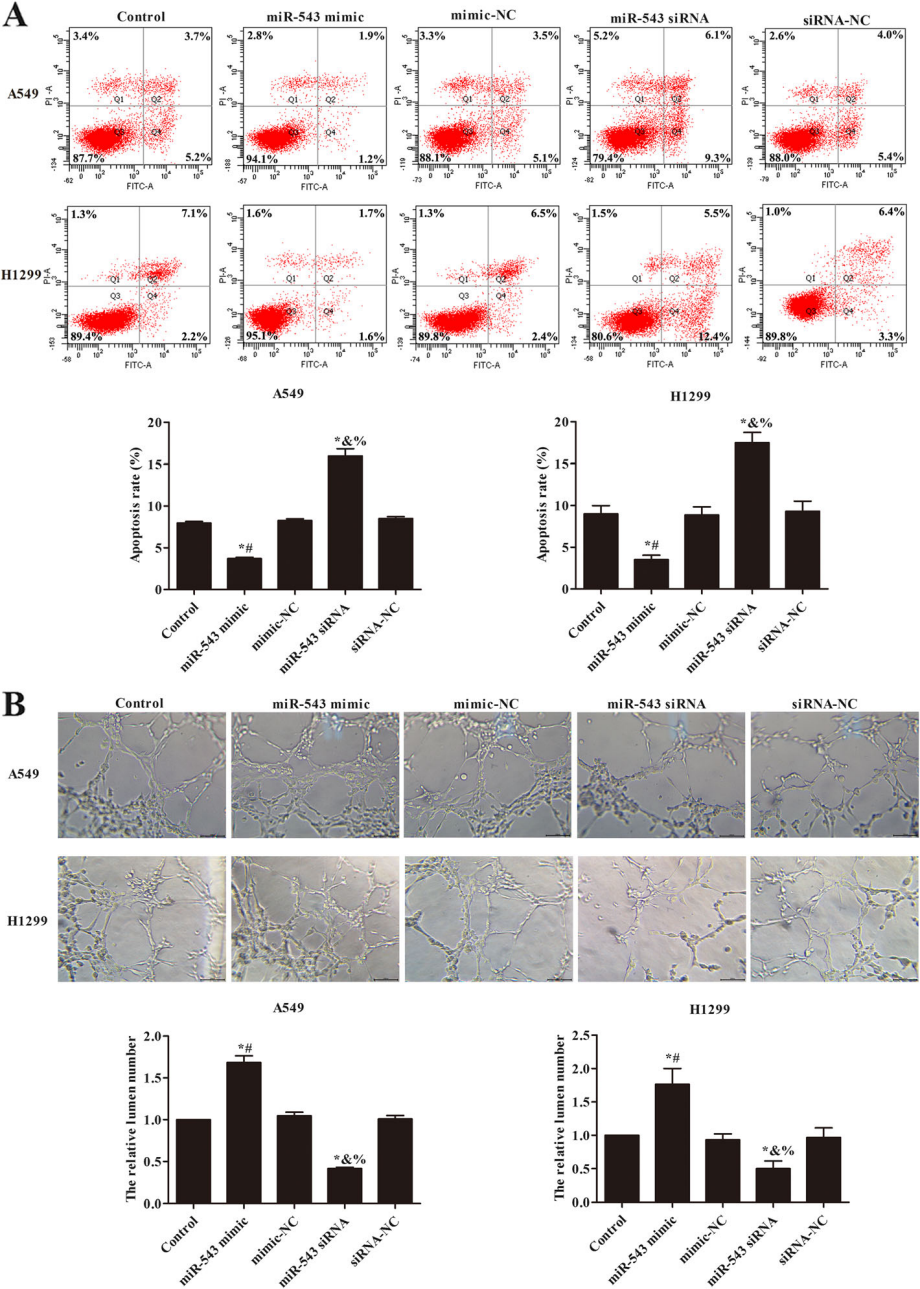
miR-543 overexpression promoted the angiogenesis of A549 and H1299 cells. **a** The effect of miR-543 on the apoptosis of A549 and H1299 cells was detected by flow cytometry. **b** Tube formation assay detected the effect of miR-543 on the tube formation of A549 and H1299 cells

The cells apoptosis of A549 and H1299 were further analyzed in Fig. 2a. Compared with control group, the apoptosis rate of A549 and H1299 significantly reduced in miR-543 mimic group (*p* < 0.05), nevertheless, the apoptosis rate of the two cell lines were obviously increased in miR-543 siRNA group (*p* < 0.05). The angiogenesis of A549 and H1299 was investigated using tube formation assay (Fig. 2b). We found that the relative lumen number was notably raised in miR-543 mimic group, but decreased in miR-543 siRNA group compared with control group (*p* < 0.05). These results revealed that miR-543 overexpression reduced the cells apoptosis and promoted cells angiogenesis in NSCLC.

### miR-543 overexpression up-regulated the expression of MTA1 in A549 and H1299 cells

**Fig. 3:**
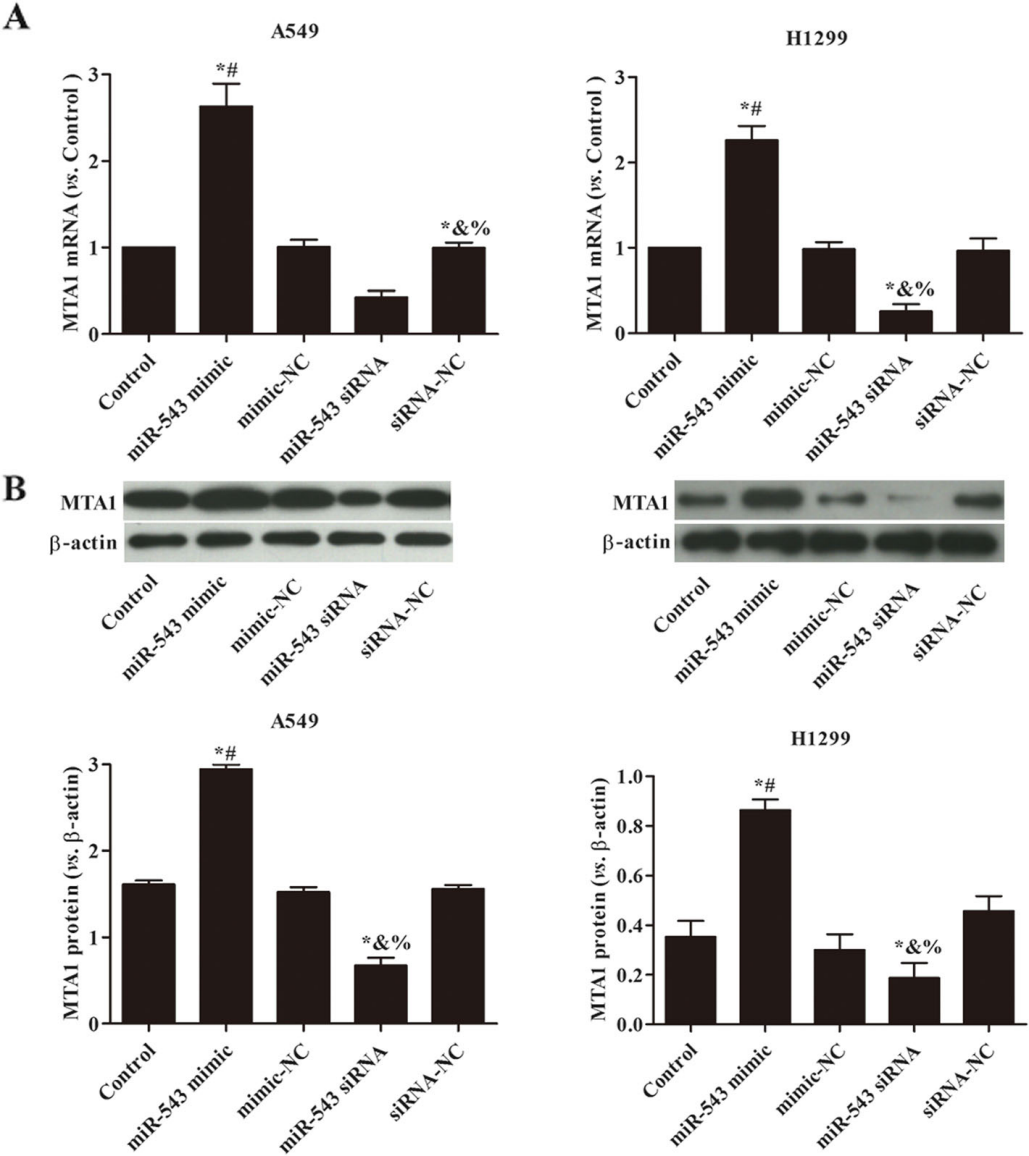
miR-543 overexpression up-regulated the expression of MTA1 in A549 and H1299 cells. **a** The expression of MAT1 mRNA in each group was detected by qRT-PCR; **b** The expression of MAT1 protein in each group was detected by western blot. Compared with control group, ^*^p < 0.05; Compared with mimic-NC, ^#^*p* < 0.05; Compared with siRNA-NC, ^&^*p* < 0.05; Compared with miR-543 mimic, ^%^*p* < 0.05

The effect of miR-543 on MTA1 expression in A549 and H1299 cells was researched by qRT-PCR and western blot, respectively (Fig. 3). It was found that, compared with control group, the overexpression of miR-543 could significantly up-regulate the MTA1 expression, while silence of miR-543 could significantly reduce the MTA1 expression (*p* < 0.05). This suggested that miR-543 promoted NSCLC progress via regulating MTA1.

### MTA1 is a downstream target gene of miR-543 in A549 and H1299

**Fig. 4:**
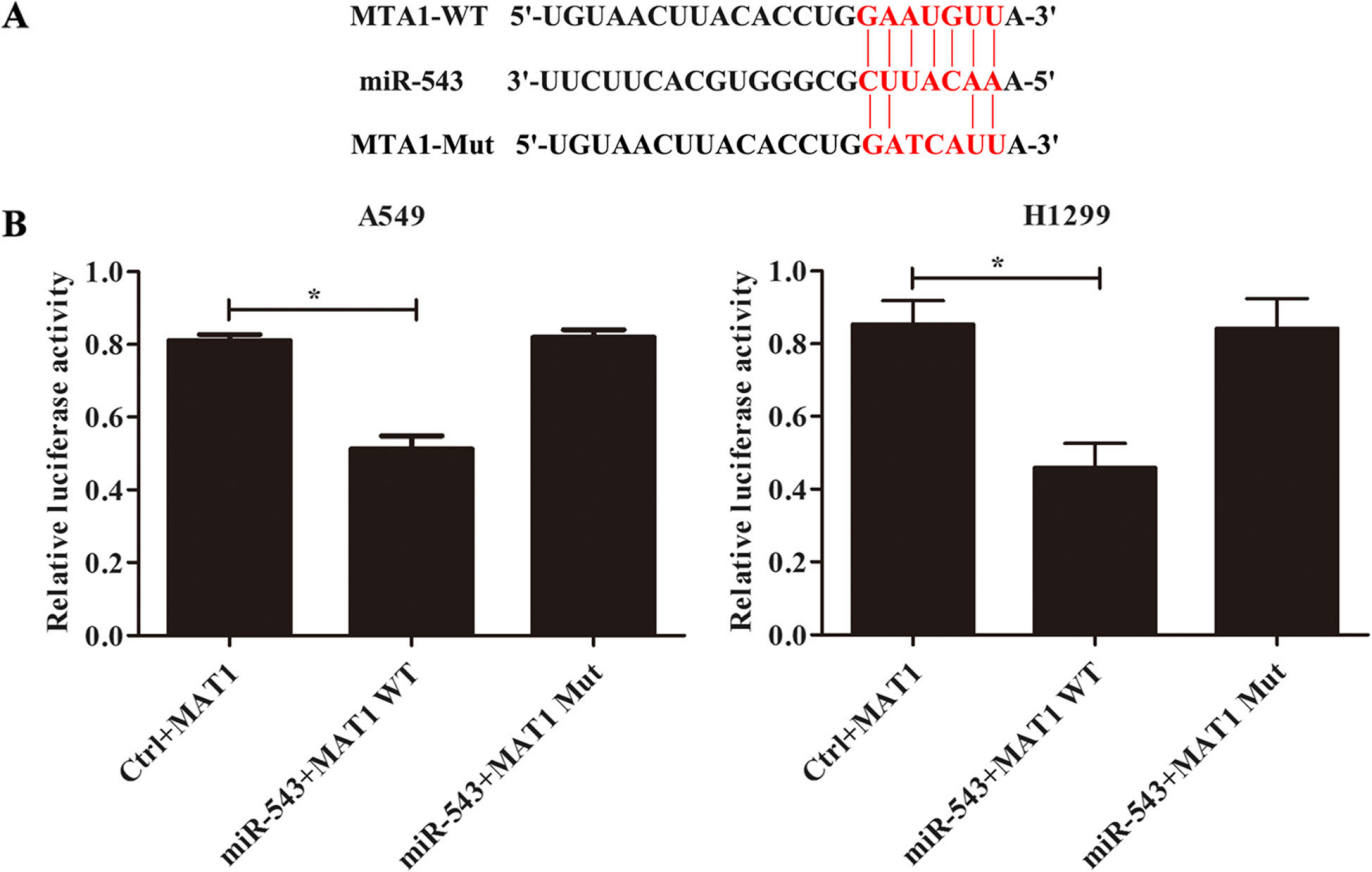
MTA1 is a downstream target gene of miR-543. **a** Mutations of 3’UTR sequence of MTA1 gene in mir-543 seed region; **b** The relative luciferase activity of MTA1 wild-type and mutant 3’UTR was analyzed by dual luciferase reporter assay. ^*^p < 0.05

As showed in Fig. 4a, bioinformatics retrieval confirmed that MTA1 was the target of miR-543. To further verify whether miR-543 targets MTA1 in A549 and H1299 cells, a dual luciferase reporter system was used. The results showed that miR-543 reduced the luciferase activity of MTA1 containing WT 3’utr but did not reduce the luciferase activity of MTA1 containing Mut 3’utr in A549 and H1299 cells (Fig. 4b, *p* < 0.05).

### miR-543 overexpression promoted cells proliferation of A549 and H1299 via regulating MTA1

**Fig. 5:**
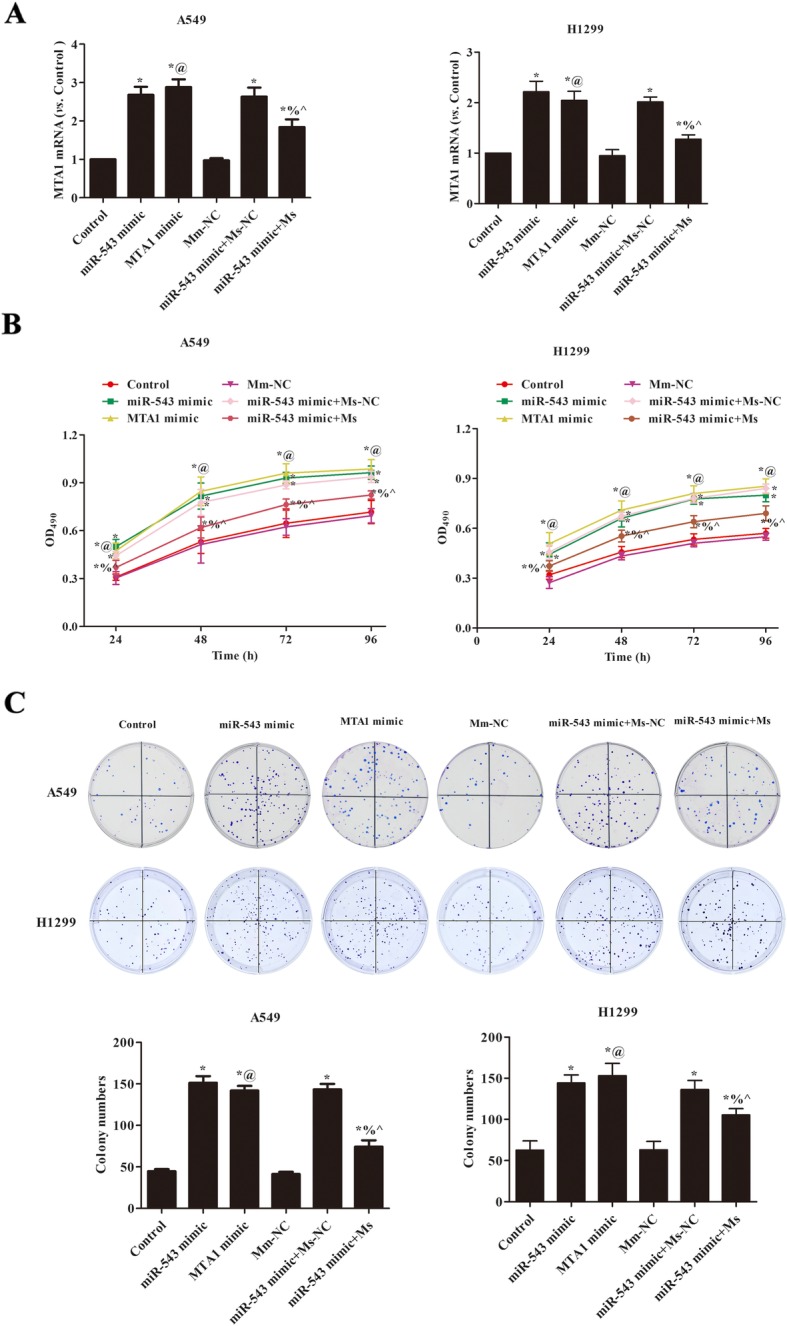
miR-543 overexpression increased the proliferation of A549 and H1299 cells via regulating MTA1. **a** The levels of MTA1 mRNA in each group were analyzed by qRT-PCR; **b** MTT detected the effect of miR-543 overexpression on the proliferation of A549 and H1299 cells by regulating MTA1. **c** Plate cloning assay was used to detect the effect of miR-543 overexpression on the proliferation of A549 and H1299 cells by regulating MTA1. Compared with control group, ^*^p < 0.05; Compared with miR-543 mimic, ^%^*p* < 0.05. Compared with Mm-NC, ^@^*p* < 0.05; Compared with miR-543 mimic+ Ms-NC, _^_*p* < 0.05

In order to verify the interaction between miR-543 and MTA1 on proliferation in NSCLC cells, we constructed MTA1 overexpresion and lowexpression in A549 and H1299. As showed in the Fig. 5a, the transfection efficiency of MTA1 was analyzed by qRT-PCR. The results showed that MTA1 mimic and siRNA were successfully transfected. Further the effects of miR-543 on proliferation via regulating MTA1 in both cells were studied. The cells abilities were significantly increased in MTA1 mimic and miR-543 mimic group, but MTA1 siRNA obviously weakened the effect of miR-543 (*p* < 0.05, Fig. 5b). For clone assay (Fig. 5c), transfection of MTA1 mimic or miR-543 mimic markedly raised the colony numbers in A549 and H1299 cells, while transfection of MTA1 siRNA notably reduced the role of miR-543 mimic (*p* < 0.05, Fig. 5c). This suggested that miR-543 overexpression promoted NSCLC proliferation via regulating MTA1.

### miR-543 overexpression promoted the angiogenesis of A549 and H1299 via regulating MTA1

**Fig. 6:**
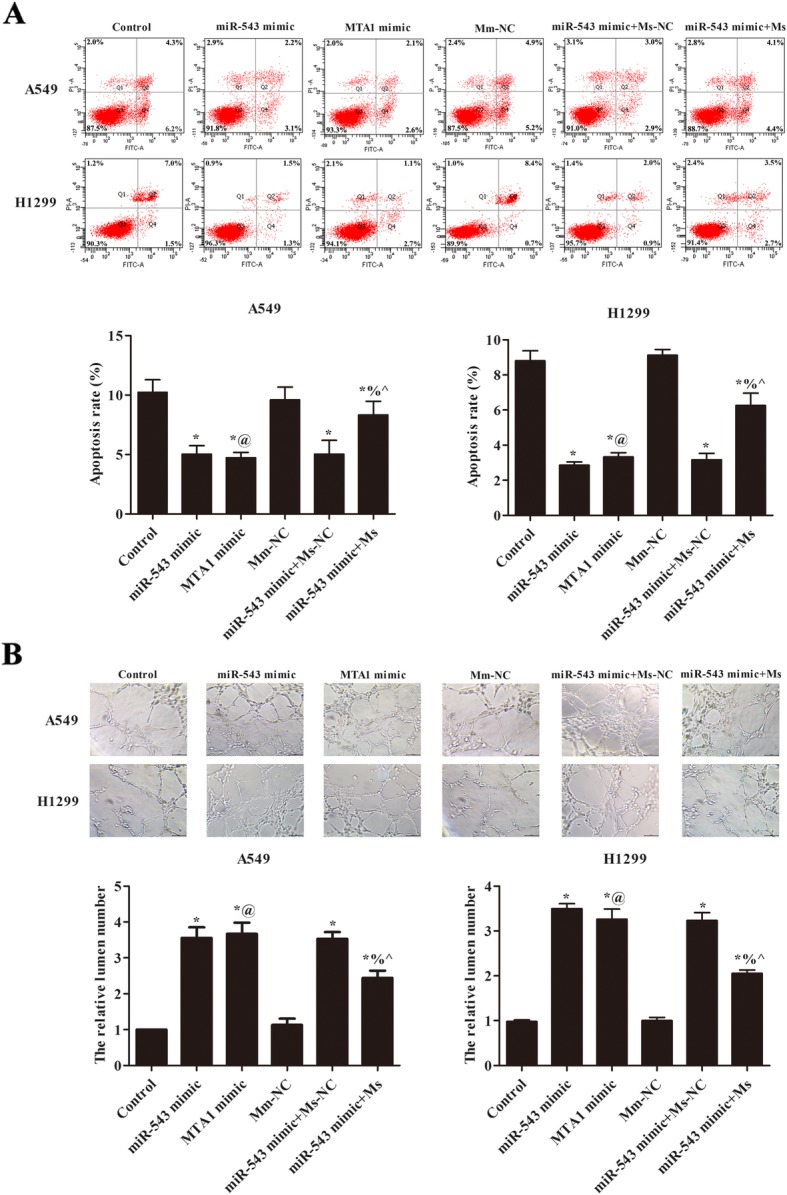
miR-543 overexpression promoted the angiogenesis of A549 and H1299 cells via regulating MTA1. **a** Flow cytometry was used to detect the effect of miR-543 overexpression on the apoptosis of A549 and H1299 cells by regulating MTA1. **b** Tube formation assay verified the effect of miR-543 overexpression on the tube formation of A549 and H1299 cells by regulating MTA1. Compared with control group, ^*^p < 0.05; Compared with miR-543 mimic, ^%^*p* < 0.05. Compared with Mm-NC, ^@^*p* < 0.05; Compared with miR-543 mimic+ Ms-NC, ^^^*p* < 0.05

The apoptosis rate of each group in A549 and H1299 was analyzed by flow cytometry (Fig. 6a). Transfection of miR-543 mimic or MTA1 mimic significantly inhibited cell apoptosis, and transfection of MTA1 siRNA notably reduced the effect of miR-543 mimic (*p* < 0.05). The effect of miR-543 on angiogenesis of A549 and H1299 via regulating MTA1 was analyzed (Fig. 6b). The relative lemen numbers were notably raised after transfection of miR-543 mimic or MTA1 mimic, and transfection of MTA1 siRNA weakened the angiogenesis of miR-543 mimic (*p* < 0.05). These data revealed that miR-543 overexpression promoted NSCLC angiogenesis via regulating MTA1.

### miR-543 overexpression promoted tumor growth via regulating MTA1 in vivo

**Fig. 7:**
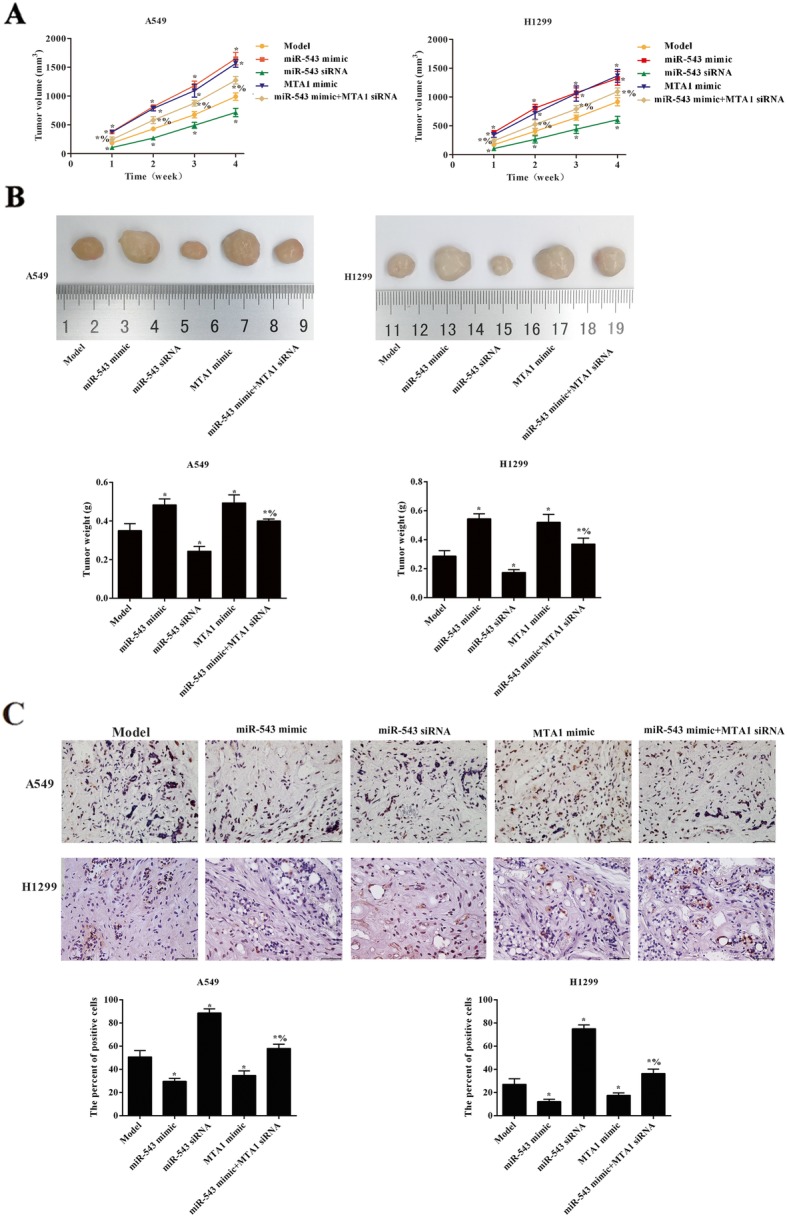
miR-543 overexpression promoted tumor growth via regulating MTA1 in tumor-bearing nude mice. **a** Tumor volume. **b** Tumor weight. **c** TUNEL was used to detect the cell apoptosis in tumor tissues of each group. Compared with model group, *p < 0.05; Compared with miR-543 mimic group, ^%^*p* < 0.05

The tumor volume and tumor weight were observed in Fig. 7a, b. The results showed that miR-543 or MTA1 overexpression promoted the growth of tumor with increase of tumor volume and weight (*p* < 0.05). After transfection with miR-543 siRNA, the tumor volume and weight were significantly lower than that of model (*p* < 0.05). Compared with the miR-543 mimic group, tumor volume and weight were reversed in the miR-543 mimic+MTA1 siRNA group (*p* < 0.05). Cell apoptosis of tumor tissue was analyzed by TUNEL (Fig. 7d). It was found that the overexpression of miR-543 or MTA1 significantly reduced the cell apoptosis, while the cell apoptosis increased after transfection with miR-543 siRNA or miR-543 mimic+MTA1 siRNA (*p* < 0.05). These revealed that miR-543 overexpression promoted tumor growth via regulating MTA1.

### miR-543 overexpression promoted tumor angiogenesis by upregulating MTA1

**Fig. 8:**
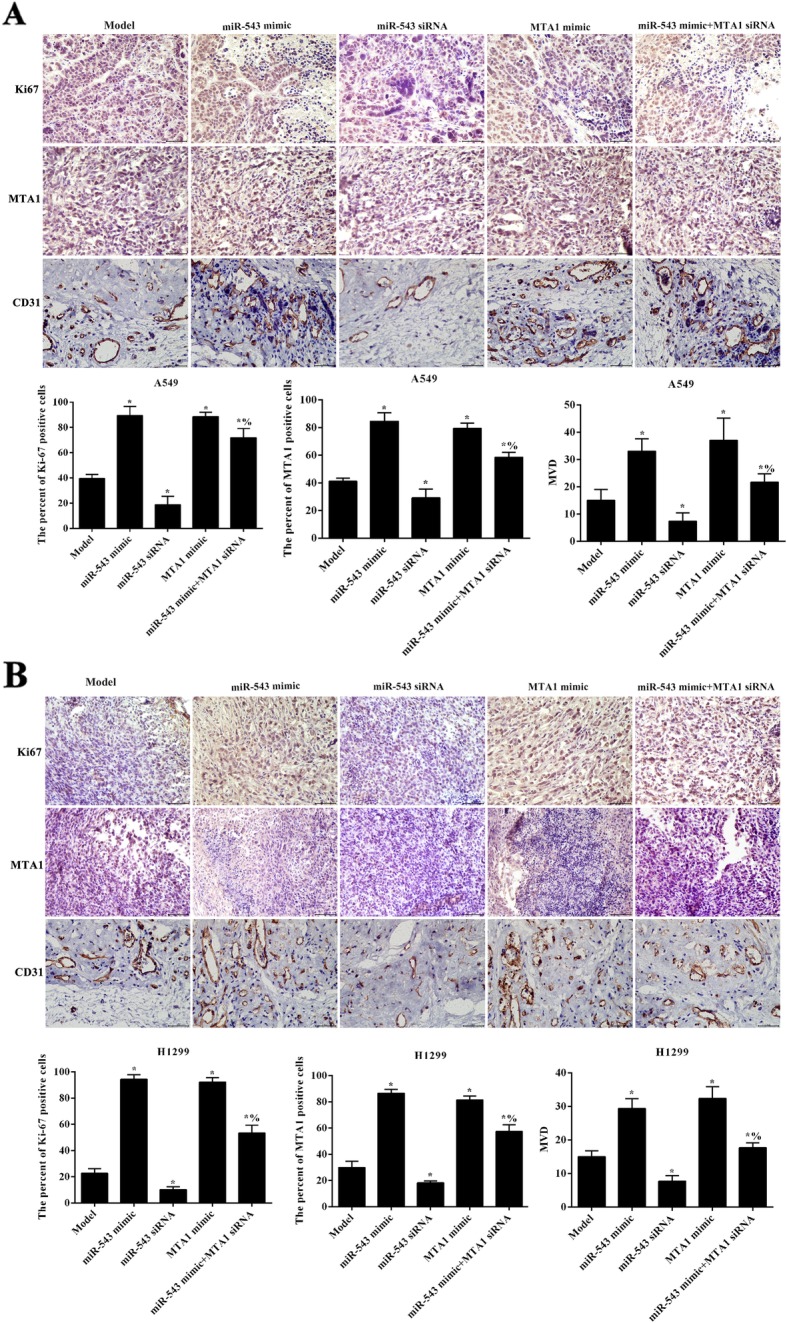
miR-543 overexpression promoted Ki67 expression and tumor angiogenesis by upregulating of MTA1. **a** Immunohistochemical analysis of Ki67, MTA1 and CD31 expression in tumor tissues of A549 cells transfection; **b** Immunohistochemical analysis of Ki67, MTA1 and CD31 expression in tumor tissues of H1299 cells transfection. Compared to model group, ^*^p < 0.05; Compared with miR-543 mimic group, ^%^*p* < 0.05

Immunohistochemistry was used to analyze the effect of miR-543 on the expression of Ki67, MTA1 and CD31 proteins in tumor tissues by targeting MTA1 (Fig. 8). It was found that both miR-543 overexpression and MTA1 overexpression significantly increased the expressions of Ki67, MTA1 and CD31 (*p* < 0.05). However, these proteins expression were significantly reduced after transfection with miR-543 siRNA (*p* < 0.05). Simultaneously transfection with miR-543 mimic and MTA1 siRNA, the levels of Ki67, MTA1 and CD31 were significantly reduced compared with miR-543 mimic group (*p* < 0.05). The data indicated miR-543 overexpression increased tumor microvascular density by upregulating MTA1.

## Discussion

Our study has shown the overexpression of miR-543 could promote proliferation and angiogenesis of A549 and H1299 cells, and similar results were showed in vivo. In published studies, overexpression of miR-543 would promote the tumorigenesis of pituitary adenoma (Shen et al. 2019), renal cell carcinoma (Yang et al. 2018), gastric cancer (Xu et al. 2018), esophageal cancer (Zhao et al. 2018), and prostate cancer (Du et al. 2017), etc. However, contrary conclusions have been reported regarding the role of miR-543 in prostate cancer (Du et al. 2017). Regarding the role of miR-543 in NSCLC, we have only made a preliminary conclusion.

Studies have shown that high expression of MTA1 is usually discovered in patients with lung infiltrating adenocarcinoma, which is statistically relevant to tumor sizes, lymph node metastasis and microvascular density (Liu et al. 2018; Zhu et al. 2017). Survival analysis showed that high expression of MTA1 was negatively correlated with 5-year survival, compared to patients with lower expression of MTA1 (Li et al. 2011). MTA1 is a potential new therapeutic target for anti-angiogenesis in patients with lung infiltrating adenocarcinoma (Li et al. 2016b). These findings suggest that expression of MTA1 have clinical potential value as a progressive phenotype indicator.

In addition, previous studies explore that MTA1 is closely related to tumor angiogenesis, including NSCLC (Andishehtadbir et al. 2015; Wang et al. 2019). MTA1 contributes to the formation of new blood vessels in residual tumors. Cell experiments further demonstrated that overexpression of MTA1 enhanced tube formation and new blood vessels in chick embryos (Xue et al. 2017). In vivo experiments also confirmed that MVD and tumor growth rate decreased after MTA1 silencing in NSCLC, and 70% of the nude mice survived for more than 30 days (Wang et al. 2019). The expression of MTA1, CD34, vascular endothelial growth factor (VEGF), alpha smooth muscle actin (α-SMA), and HIF-1-α in the lungs of knockout and wild-type mice was examined to study the effect of MTA1 on alveolar capillary formation in mice (Qin et al. 2017). In which, the number of alveolar capillaries was decreased in MAT1 knockount mice, and the expressions of HIF-1-α and VEGF were decreased in the lungs. Consistent with these published reports, this study showed overexpression of MTA1 enhanced the proliferation and angiogenesis in vitro and in vivo*.*

It has been reported that miR-543 was negatively correlated with the expression of MTA1 in clinical samples of colorectal cancer and the direct interaction between miR-543 and MTA1 was confirmed by double luciferase experiment at the molecular level (Fan et al. 2016). This is consistent with the previous report, that MTA1 is a downstream regulatory target of miR-543 and miR-543 overexpression could up-regulate MTA1 expression in NSCLC. Many limitations were existed in this research. The mechanism of miR-543 in NSCLC need to further study. In addition, the clinical research between miR-543 and MTA1 is also research.

## Conclusion

In summary, the present study showed that miR-543 overexpression promoted proliferation and angiogenesis in NSCLC and the MTA1 was a target gene of miR-543. The findings could provide a better understanding the mechanism of miR-543 on regulation of NSCLC.

## Data Availability

Data is not available, you can contact Xudong Tian by email if needed, zhangding553977@126.com

## Abbreviations

NSCLC: non-small cell lung cancer
MTA1: metastasis associated protein 1
MVD: microvessel density
VEGF: vascular endothelial growth factor
